# *Entamoeba* species infection in patients seeking treatment for diarrhea and abdominal discomfort in Mukuru informal settlement in Nairobi, Kenya

**DOI:** 10.1016/j.fawpar.2021.e00122

**Published:** 2021-03-29

**Authors:** Erastus Mulinge, Cecilia Mbae, Benjamin Ngugi, Tabitha Irungu, Elizabeth Matey, Samuel Kariuki

**Affiliations:** Centre for Microbiology Research, Kenya Medical Research Institute, P.O. Box 19464 00202, Nairobi, Kenya

**Keywords:** *Entamoeba* species, Diarrhea, Abdominal discomfort, Mukuru informal settlement, Nairobi, Kenya

## Abstract

*Entamoeba histolytica* is the only pathogenic species of the *Entamoeba* genus and is morphologically identical to *E. dispar*/*E. moshkovskii* (*Entamoeba* complex) hence cannot be microscopically differentiated. The other *Entamoeba* spp. found in humans (*E. hartmanni, E. polecki,* and *E. coli*) can be differentiated morphologically from this *Entamoeba* complex. However, some of their morphologic features overlap making differential diagnosis difficult. This study aimed at determining the occurrence of *Entamoeba* spp. in patients seeking treatment for diarrhea and/or abdominal discomfort at two clinics in Mukuru informal settlement in Nairobi, Kenya. Faecal samples were collected from 895 patients, examined microscopically following direct wet smear and formal-ether concentration methods. *Entamoeba* spp. positive faecal samples were subjected to DNA extraction and species-specific nested polymerase chain reaction of the 18S ribosomal RNA (rRNA). By microscopy, *Entamoeba* spp. cysts or trophozoites were detected in 114/895 (12.7%, 95% Confidence Interval (CI) 10.6–15.1) faecal samples. By nested PCR, the prevalence was: *E. histolytica* (7.5%, 95% CI 5.9–9.4, 67/895) and *E. dispar* (8.2%, 95% CI 6.5–10.2, 73/895). Among the *Entamoeba* spp. complex positive samples, nested PCR detected *E. coli* and *E. hartmanni* DNA in 63/114 (55.3%) and 37/114 (32.5%), samples respectively. Among the *E. histolytica*/*E. dispar* PCR negative samples (32.5%), 21 (18.4%) contained cysts of either *E. coli* (19) or *E. hartmanni* (2) by nested PCR. *Entamoeba* spp. infections were most common among participants aged 21–30 years; however it was not significant (*P* = 0.7). *Entamoeba* spp. infections showed an inverse relationship with diarrhea being most common among participants without diarrhea (*P* = 0.0). The difference was significant for *E. histolytica* (*P* = 0.0) but not significant for *E. dispar* (*P* = 0.1). Only *E. dispar* infections were significantly associated with sex (*P* = 0.0). This study highlights the need for differentiation of *E. histolytica* from other *Entamoeba* spp. by molecular tools for better management of amoebiasis.

## Highlights

• Identified four *Entamoeba* species (*E. histolytica, E. dispar, E. coli* and *E. hartmanni*)
• *Entamoeba* spp. infection were most common in participants aged 21–30 years.
• *Entamoeba* spp. infection were inversely related to diarrhea.
• *E. coli* and *E. hartmanni* were detected in faecal samples containing *Entamoeba* spp. complex.

## 1 Introduction

The protozoan *Entamoeba histolytica* is the causative agent of amoebiasis, where patients are asymptomatic or may present with amoebic dysentery and liver abscess among other symptoms. The burden of amoebiasis is high in developing countries due to poor sanitary conditions, low socioeconomic status and non-hygienic practices (Shirley et al., 2018). An estimated 50 million people worldwide are infected with *E. histolytica* annually leading to death of 100,000 people (WHO, 1997). The genus *Entamoeba* consists of many species, seven of which colonize the human intestinal lumen, namely *Entamoeba histolytica*, *Entamoeba dispar*, *Entamoeba moshkovskii*, *Entamoeba polecki*, *Entamoeba coli*, *Entamoeba hartmanni,* and *Entamoeba bangladeshi* (Fotedar et al., 2007; Royer et al., 2012). *Entamoeba histolytica* cause amoebic colitis and extra-intestinal disease in humans, while patients infected with either *E. dispar* and/or *E. moshkovskii* have presented with gastrointestinal symptoms (Fotedar et al., 2008; Ximenez et al., 2010). The other *Entamoeba* species are commensal parasites in humans (Stanley Jr., 2003).

Microscopy has been the traditional method for diagnosis of *E. histolytica/E. dispar* in stool despite having low sensitivity (Gonzalez-Ruiz et al., 1994). The sensitivity of microscopy is further reduced by the periodic release of cysts that necessitates the examination of multiple faecal samples in subsequent days (Nazer et al., 1993). Additionally, pathogenic *E. histolytica* is indistinguishable in its cysts and trophozoite stages from non-pathogenic *E. dispar* and *E. moshkovskii* (WHO, 1997; Ali et al., 2003). The diagnosis is also complicated by the presence of other commensal *Entamoeba* spp. (*E. hartmanni, E. coli*, and *E. polecki*) with overlapping morphologic features (Tanyuksel and Petri Jr., 2003). Therefore, there is need for sensitive and accurate diagnostic tools to inform the management of amoebiasis and reduce unnecessary treatment for non-pathogenic *Entamoeba* spp. (WHO, 1997; Pritt and Clark, 2008). Polymerase chain reaction (PCR), is one of such tools that can differentiate *E. histolytica* from *E. dispar*/*E. moshkovskii* and other species of the *Entamoeba* genus. The *Entamoeba* spp. 18S rRNA gene exists as a multicopy loci and exhibits genetic variation and therefore a good target for detection and differentiation of members of this genus (Cruz-Reyes et al., 1992; Troll et al., 1997; Bhattacharya et al., 1998). The development and application of a nested multiplex PCR based on 18S rRNA has contributed greatly into epidemiology of amoebiasis in many regions of the world (Khairnar and Parija, 2007).

Worldwide more than half of the population live in urban areas and this has resulted in exponential growth of informal settlements. In Kenya the informal settlements are home to more than 71% of the urban population (WHO and UN-Habitat, 2010). They are densely populated, lack clean water and have inadequate sanitation, poor waste management and drainage (APHRC, 2014). These conditions favour parasite transmission and put the residents at risk of acquiring infectious diseases with high morbidity and mortality (Brooker et al., 2006). Indeed the Mukuru informal settlements are hot spots for infectious diseases such as multidrug resistant nontyphoidal *Salmonella* and intestinal protozoans according to findings of Kariuki et al. (2019) and Mbae et al. (2013), respectively.

Although *E. histolytica/E. dispar* is one of the most common parasitic cause of diarrheal diseases in Kenya, besides *Cryptosporidium* species and *Giardia lamblia* (Gatei et al., 2006; Mbae et al., 2013). The epidemiology of amoebiasis is poorly understood owing to the fact that previous studies employed microscopy for diagnosis and could not distinguish pathogenic *E. histolytica* from other commensal *Entamoeba* spp. Therefore, the actual prevalence of *E. histolytica* in those studies is still unknown since microscopy was shown to overestimate the prevalence of *E. histolytica* when molecular tools were applied in studies across African countries (Kebede et al., 2003; Ben Ayed et al., 2008; Efunshile et al., 2015; Yimer et al., 2017). In addition, there is limited data on the occurrence of other *Entamoeba* spp. (*E. hartmanni, E. coli, E. polecki*) (Matey et al., 2016) and how their presence could easily mislead the diagnosis of *E. histolytica*. The present study reports the prevalence of *Entamoeba* spp. in patients seeking treatment for diarrhea and/or abdominal discomfort at two clinics in Mukuru informal settlement in Nairobi, Kenya.

## 2 Materials and methods

### 2.1 Study design and study sites

**Fig. 1:**
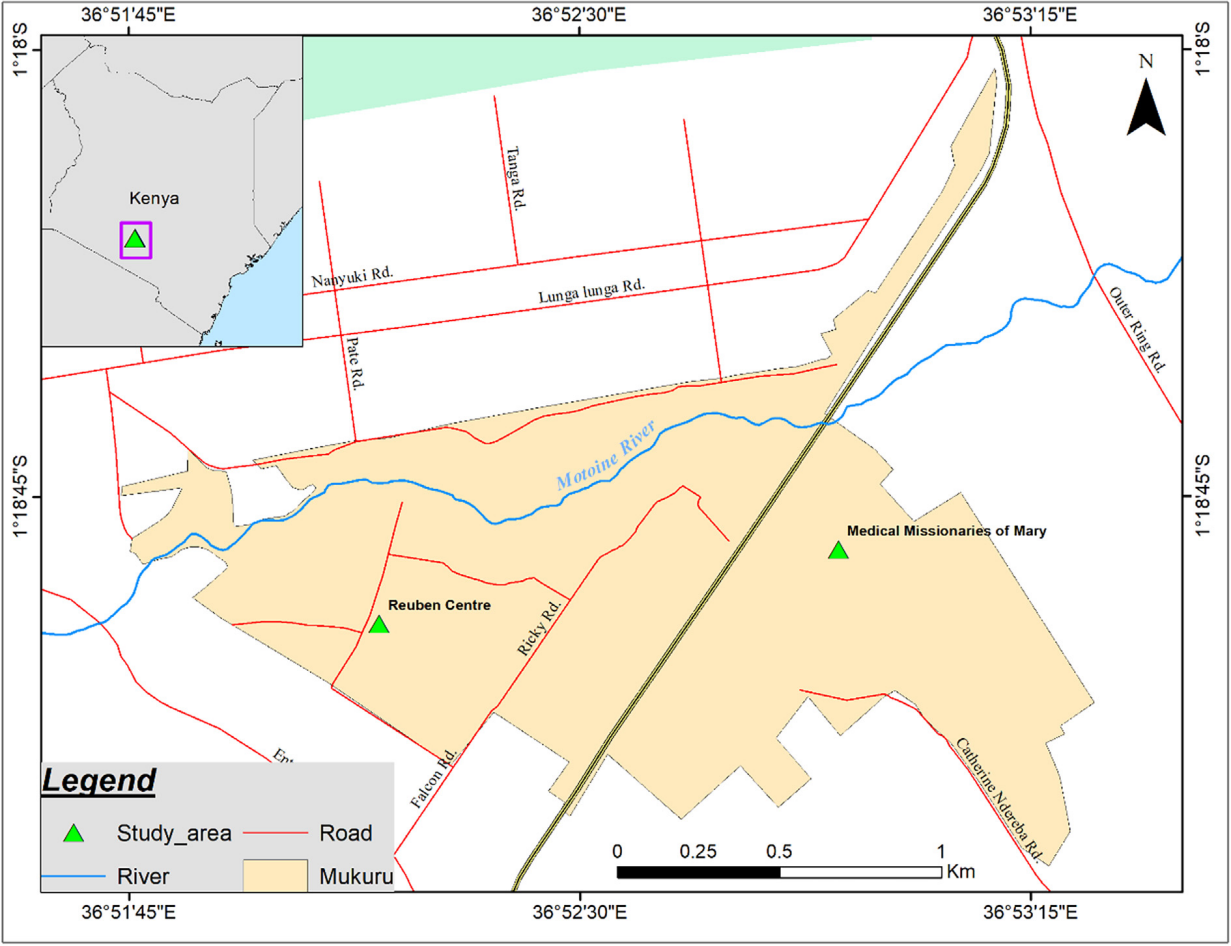
A map of Mukuru informal settlement showing the Reuben Centre and Medical Missionaries of Mary clinics.

This was a cross-sectional study carried out at two clinics (Reuben Centre and Medical Missionaries of Mary) within Mukuru informal settlement in Nairobi, Kenya (Fig. 1). Mukuru slum is one of the urban informal settlements in Nairobi city and is subdivided into 8 villages. The Reuben Centre (1° 18′ 57” S, 36° 52′ 10″ E) and Medical Missionaries of Mary clinics (MMM) (1° 18′ 50” S, 36° 52′ 55″ E) are located in Mukuru Kwa Reuben and Mukuru Kwa Njenga villages respectively. The villages are characterized by poor sanitary conditions, poor drainage systems, shortage of clean drinking water and improper waste management factors that are likely to enhance transmission of enteric infections such as amoebiasis.

### 2.2 Specimen collection, processing and microscopy examination

The majority of the patients seeking treatment in these clinics reside within Mukuru informal settlement. Fresh faecal samples were collected (February – May 2013) from individual patients of all ages presenting with gastrointestinal symptoms (diarrhea, abdominal pain, bloody stool). The faecal samples were examined macroscopically for consistency, mucus and blood and processed by direct wet smear and formal-ether sedimentation techniques, iodine staining and examined microscopically for the presence of cysts or trophozoites of *Entamoeba* spp. and other intestinal parasites (Cheesbrough, 2009). Stool samples found positive for *Entamoeba* spp. cysts or trophozoites were aliquoted and preserved in 2.5% potassium dichromate at 4 °C until further analysis. For detection of *Cryptosporidium* and other protozoa, faecal samples were subjected to Modified Ziehl-Neelsen staining technique and examined by microscopy (Casemore, 1991).

### 2.3 *Extraction of* Entamoeba spp. *genomic DNA*

Genomic DNA was extracted from 114 stool samples microscopically positive for *E. histolytica/E. dispar*. Briefly 200 μl of faecal suspension from each *Entamoeba* spp. positive sample was washed five times with triple-distilled water by centrifugation. DNA was purified using QIAamp® DNA Stool Mini Kit (Qiagen, Hilden, Germany) with slight modifications of the manufacturer's protocol. The cysts were lysed by adding 1.4 ml of ASL buffer and subjected to five cycles of freeze (−80 °C for 30 min) and thaw (80 °C for 15 min). The DNA was eluted in 50 μl of elution buffer and stored at −20 °C before used in PCR reactions. *Entamoeba* spp. control DNA was obtained from the Department of Parasitology, Medical School, Kanazawa University, Japan courtesy of Dr. Tokoro Masaharu.

### 2.4 Nested polymerase chain reaction based on 18S ribosomal RNA gene

A nested PCR targeting 18S ribosomal RNA gene was performed for detection and differentiation of *E. histolytica*, *E. dispar* and *E. moshkovskii* according to Khairnar and Parija (2007) with slight modifications. The primary PCR which is *Entamoeba* genus-specific used forward primer E-1 and reverse primer E-2 (Table 1). The PCR assay was carried out in a 25 μl reaction volume consisting of 1 × 10 mM Tris-HCl (pH 8.8), 50 mM KCl, 2 mM MgCl_2_, 200 μM of each dNTPs, 0.625 units of DreamTaq™ Green DNA Polymerase, (Thermo Fisher Scientific, Massachusetts, USA), 0.25 μM of each primer and 2.5 μl of DNA template. The cycling conditions included an initial denaturation at 94 °C for 5 min, followed by 40 cycles of 94 °C for 30 s, 58 °C for 30 s, 72 °C for 1 min and a final extension at 72 °C for 7 min. The nested PCR for the *Entamoeba* complex differentiation were done individually instead of using multiplex PCR. Amplification was achieved using primer sets EH-1 and EH-2 for *E. histolytica*; ED-1 and ED-2 for *E. dispar*; Mos-1 and Mos-2 for *E. moshkovskii* (Table 1) as described by Khairnar and Parija (2007). In the secondary amplification reagent concentrations and cycling conditions were similar to the first PCR except that 2.5 μl of the primary PCR product was used as DNA template and different annealing temperatures were used for each species, 52 °C for *E. dispar*, 50 °C for *E. histolytica* and 48 °C for *E. moshkovskii* (Table 1). To illustrate how other *Entamoeba* spp. beside *E. histolytica/E. dispar/E. moshkovskii* complex might interfere with microscopy diagnosis of amoebiasis, another nested PCR was performed on the faecal samples containing this complex but targeting *E. coli* and *E. hartmanni* DNA as previously described by Matey et al. (2016). For the primary PCR universal *Entamoeba* genus-specific forward (TN21) and reverse (TN14) primers were used. In the secondary PCR species-specific forward primers MA67 and MA113 for *E. hartmanni* and *E. coli* respectively, the reverse primer TA28 was used for both species (Table 1). The secondary PCR products were subjected to electrophoresis in 2% agarose gels stained with ethidium bromide and visualized on a UV-transilluminator.

**Table 1:** Primers for detection of *Entamoeba* species.

Primer	Genus/species	Sequence (5′-3′)	Annealing temperature	Reference
E-1	*Entamoeba*	TAAGATGCAGAGCGAAA	58 °C	(Khairnar and Parija, 2007)
E-2	*Entamoeba*	GTACAAAGGGCAGGGACGTA	58 °C	(Khairnar and Parija, 2007)
EH-1	*E. histolytica*	AAGCATTGTTTCTAGATCTGAG	50 °C	(Khairnar and Parija, 2007)
EH-2	*E. histolytica*	AAGAGGTCTAACCGAAATTAG	50 °C	(Khairnar and Parija, 2007)
ED-1	*E. dispar*	TCTAATTTCGATTAGAACTCT	52 °C	(Khairnar and Parija, 2007)
ED-2	*E. dispar*	TCCCTACCTATTAGACATAGC	52 °C	(Khairnar and Parija, 2007)
Mos-1	*E. moshkovskii*	GAAACCAAGAGTTTCACAAC	48 °C	(Khairnar and Parija, 2007)
Mos-2	*E. moshkovskii*	CAATATAAGGCTTGGATGAT	48 °C	(Khairnar and Parija, 2007)
TN21	*Entamoeba*	AAGATTAAGCCATGCATGTSKA	58 °C	(Matey et al., 2016)
TN14	*Entamoeba*	GATACCTTGTTACGACTTCTY	58 °C	(Matey et al., 2016)
MA67	*E. hartmanni*	TTGGATGTAGAGATACATTC	54 °C	(Matey et al., 2016)
T28	*E. hartmanni*	CACTATTGGAGCTGGAATTAC	54 °C	(Matey et al., 2016)
MA113	*E. coli*	GCCAAGAGAATTGTAGAAATCG	54 °C	(Matey et al., 2016)
T28	*E. coli*	CACTATTGGAGCTGGAATTAC	54 °C	(Matey et al., 2016)

### 2.5 Ethical approval

The study protocol (SSC. No. 2258) was reviewed and approved by the Scientific and Ethics Review Unit (SERU) at KEMRI and by the management of Mukuru clinics (Reuben Centre and Medical Missionaries of Mary). Verbal consent was sought from all study participants before they were enrolled.

### 2.6 Data analysis

The data was entered in Microsoft Excel 2013 and transferred into STATA version 12.0 (STATA Corporation, College Station, Texas, USA) for analysis. The chi square (χ^2^) test was used to determine the potential correlation between age, sex of the study participants, centre and stool constituency with *Entamoeba* infections. The difference was considered significant when the *P* value was ≤0.05.

## 3 Results

### 3.1 Demographic information

A total of 895 participants were recruited into the study, 470 from the Medical Missionaries of Mary (MMM) and 425 from the Reuben Centre. Among the participants 439 (49.1%) were males, 426 (47.6%) females and 30 (3.4%) whose gender was not recorded (Table 2), the latter were excluded from analysis (Table 3). The ages of the study participants ranged from 2 weeks to 83 years and were stratified into 8 groups (0–5, 6–10, 11–20, 21–30, 31–40, 41–50, 51–60 and ≥ 61 years). The majority of the participants were aged 21–30 years (34.8%), followed by infants under 5 years at 16.0% while only 2 participants (0.2%) were aged 61 and above. The ages of 11 (1.2%) participants were not recorded (Table 2) and were excluded from analysis (Table 3).

**Table 2:** Demographic data and prevalence of *Entamoeba* species by age, sex and centre.

Variables	No. of subjects (%) *n* = 895	Microscopy (%) *n* = 114 (12.7)	*E. histolytica* (%) *n* = 67 (7.5)	*E. dispar* (%) *n* = 73 (8.2)	*E. coli* (%) *n* = 63 (55.3)	*E. hartmanni* (%) *n* = 37 (33.0)
Age groups (years)						
≤5	143 (16.0)	14	9	11	8	5
6-10	119 (13.3)	14	5	6	6	5
11-20	114 (12.7)	13	9	9	11	6
21-30	311 (34.8)	44	23	25	25	16
31-40	131 (14.6)	17	14	13	6	4
41-50	54 (6.0)	7	5	6	6	1
51-60	10 (1.1)	2	1	1	0	0
6≥	2 (0.2)	1	0	1	0	0
Unknown	11 (1.2)	2	1	1	1	1
Gender						
Male	439 (49.1)	50 (5.6)	27 (3.0)	28 (3.1)	26 (22.8)	15 (13.2)
Female	426 (47.6)	62 (6.9)	39 (4.4)	44 (4.9)	36 (31.6)	22 (19.3)
Unknown	30 (3.4)	2 (0.2)	1 (0.1)	1 (0.1)	1 (0.9)	0 (0.0)
Centre						
Reuben	425 (47.5)	54 (12.7)	31 (7.3)	36 (8.5)	27 (23.7)	17 (14.9)
MMM	470 (52.5)	60 (12.8)	36 (7.7)	37 (7.7)	36 (31.6)	20 (17.5)

**Table 3:** Association of *Entamoeba* species infection with age, sex, centre and stool consistency.

Variables	No. of subjects (%) n = 884	Microscopy (%) n = 112 (13.0)	*E. histolytica* (%) n = 66 (7.6)	*E. dispar* (%) n = 72 (8.0)	*E. coli* (%) n = 63 (56.3)	*E. hartmanni* (%) n = 37 (32.5)
Age groups (years)						
≤5	143 (16.2)	14	9	11	8	5
6-10	119 (13.5)	14	5	6	6	5
11-20	114 (12.9)	13	9	9	11	6
21-30	311 (35.2)	44	23	25	25	16
31-40	131 (14.8)	17	14	13	6	4
41-50	54 (6.1)	7	5	6	6	1
51-60	10 (1.1)	2	1	1	0	0
61≥	2 (0.2)	1	0	1	0	0
		χ^2^ = 5.0 *P* = 0.7	χ^2^ = 4.6 *P* = 0.7	χ^2^ = 7.5 *P* = 0.4		
Gender	n = 865					
Male	439 (50.8)	50 (11.4)	27 (6.2)	28 (6.4)	26 (41.3)	15 (40.5)
Female	426 (49.3)	62 (14.6)	39 (9.2)	44 (10.3)	36 (57.1)	22 (59.5)
		χ^2^ = 1.9 *P* = 0.2	χ^2^ = 2.8 *P* = 0.1	χ^2^ = 4.4 *P* = 0.0⁎		
Centre	n = 865					
Reuben	420 (48.6)	53 (12.6)	31 (7.4)	36 (8.6)	27 (24.1)	17 (15.2)
MMM	445 (51.6)	59 (13.3)	35 (7.9)	36 (8.1)	36 (32.1)	20 (17.9)
		χ^2^ = 0.1 *P* = 0.8	χ^2^ = 0.1 *P* = 0.8	χ^2^ = 0.1 *P* = 0.8		
Consistency	n = 865					
Diarrhoeic	534 (61.7)	58 (51.8)	32 (48.5)	38 (52.8)	24 (21.4)	16 (14.3)
Non-diarrhoeic	331 (38.3)	54 (48.2)	34 (51.5)	34 (47.2)	39 (34.8)	21 (18.8)
		χ^2^ = 5.4 *P* = 0.0⁎	χ^2^ = 5.3 *P* = 0.0⁎	χ^2^ = 2.7 *P* = 0.1		

⁎ *P*-value statistically significant.

### 3.2 Microscopy examination

*Entamoeba* cysts or trophozoites were detected in 114 (12.7% 95% CI 10.6–15.1) participants. The prevalence of *Entamoeba* spp. infection was higher in MMM than in Reuben Centre (13.3 *vs* 12.6% respectively), however the difference was not significant (χ^2^ = 0.1, *P* = 0.8). *Entamoeba* spp. infection were more common in female participants compared to males (14.6% *vs* 11.4%), however this difference was not statistically significant (χ^2^ = 1.9, *P* = 0.2) (Table 3). Other intestinal parasites detected included *Giardia lamblia* (2.0%), *Ascaris lumbricoides* (1.2%), *Hymenolepis nana* (0.5%), *Chilomastix mesnili* (0.2%), *Cryptosporidium* spp., (0.2%), *H. diminuta* (0.1%) and *Cyclospora* spp. (0.1%).

### 3.3 Nested PCR assays

*Entamoeba* spp. were detected in 98/114 (86.0%) faecal samples by nested PCR. The prevalence of *Entamoeba* spp. was (7.5%, 95% CI 5.9–9.4, 67/895) for *E. histolytica* and (8.2%, 95% CI 6.5–10.2, 73/895) for *E. dispar* (Table 2). The prevalence of *E. histolytica* (7.9 *vs* 7.4) and *E. dispar* (8.1 *vs* 8.6) was comparable between the two clinics (Table 3). Among the *Entamoeba* spp. complex positive samples (114), nested PCR detected *E. coli* and *E. hartmanni* DNA in 63/114 (55.3%) and 37/114 (32.5%) samples respectively (Table 2). This study did not detect *E. moshkovskii*, while *E. histolytica*/*E. dispar* mixed infections were detected in 7.0% of the faecal samples. Mixed infection with all four *Entamoeba* spp. was detected in 15 (16.8%) faecal samples. Among the *E. histolytica*/*E. dispar* PCR negative samples (32.5%), 21 (18.4%) of them contained cysts of either *E. coli* (19) or *E. hartmanni* (2) by nested PCR. *Entamoeba* spp. infections, including *E. histolytica* were highest in the age group 21–30 years. The age groups 0–5 and 31–40 years also recorded high infections for all the *Entamoeba* spp., while participants aged over 41 years had the least infections (Table 2). None of the *Entamoeba* spp. infections were significantly associated with the age of the participants (Table 3). Female participants were more infected by all the *Entamoeba* spp. compared to males: *E. histolytica* (9.2% *vs* 6.2%) and *E. dispar* (10.3% *vs* 8.3%). The difference was only significant for *E. dispar* (χ^2^ = 4.4, *P* = 0.0) but not for *E. histolytica* (χ^2^ = 2.8, *P* = 0.1) (Table 3).

### 3.4 Correlation of faecal samples consistency with the presence of Entamoeba *species*

Majority of the participants presented with diarrhea (watery, mucoid, loose stool) 534 (61.7%) against 331 (38.2%) without (formed stool). *Entamoeba* spp. infection showed an inverse relationship with diarrhea, being most common among participants without diarrhea (χ^2^ = 5.4, *P* = 0.0). The difference was significant for *E. histolytica* (χ^2^ = 5.3, *P* = 0.0), but not significant for *E. dispar* (χ^2^ = 2.7, *P* = 0.1). A total of 31 (3.5%) faecal samples contained blood, among these 4 bloody mucoid samples were positive for *E. histolytica* by PCR.

## 4 Discussion

This study reports the presence of *E. histolytica, E. dispar, E. coli* and *E. hartmanni* in patients seeking treatment for diarrhea and/or abdominal discomfort in two clinics in Mukuru informal settlement in Nairobi, Kenya. Several microscopy-based epidemiological studies in Kenya failed to differentiate *E. histolytica* from *E. dispar*/*E. moshkovskii* complex and other *Entamoeba* spp. (Chunge et al., 1991; Joyce et al., 1996; Gatei et al., 2006; Nyarango et al., 2008; Nguhiu et al., 2009; Kamau et al., 2012; Kipyegen et al., 2012; Mbae et al., 2013; Obala et al., 2013). As it is the usual practice in Kenya, these patients were treated indiscriminately using antiamoebic drugs. This study therefore, highlights the importance of differentiating of *E. histolytica* from non-pathogenic *Entamoeba* spp. before treatment of patients to avoid drug resistance.

Accurate identification and differentiation of *Entamoeba* spp. is a critical step in the management of amoebiasis as recommended by WHO (WHO, 1997). It is necessary to distinguish pathogenic *E. histolytica* infections from those of the *Entamoeba* spp. complex and other non-pathogenic species such as *E. coli*, *E. hartmanni* and *E. polecki* (Ali et al., 2008; Gomes Tdos et al., 2014). In this study, the presence of these commensal *Entamoeba* spp. was determined in faecal samples containing the *Entamoeba* spp. complex to illustrate how their presence complicates the diagnosis of *E. histolytica* microscopically. *Entamoeba coli* and *E. hartmanni* were identified in faecal samples containing *Entamoeba* spp. complex microscopically and from those negative for this complex by nested PCR. The detection of *E. coli* or *E. hartmanni* DNA in samples originally identified microscopically to contain cysts or trophozoites for *Entamoeba* complex but negative for this complex by nested PCR clearly shows the limitation of microscopy in diagnosis of *E. histolytica*. *Entamoeba coli* and *E. hartmanni* were also common among HIV-infected and HIV-uninfected children in western Kenya (Matey et al., 2016).

The prevalence of *Entamoeba* spp. by microscopy in the present study was in agreement with those reported previously from Kenya in children (Obala et al., 2013; Njambi et al., 2020), food-handlers (Kamau et al., 2012) and people of all age groups (Nguhiu et al., 2009), as well as in other African countries including Ethiopia, Cameroon and Nigeria (Aribodor et al., 2012; Assob et al., 2012; King et al., 2013). However, elsewhere in Kenya high prevalence of *Entamoeba* spp. was recorded in children under 5 years of age from Maasailand (Joyce et al., 1996), as a co-infection in sleeping sickness patients (Kagira et al., 2011) and among HIV-patients in Baringo County (Kipyegen et al., 2012). The true prevalence of *Entamoeba* spp. in this study and previous ones could be underestimates due to the limited sensitivity of microscopy (Gonzalez-Ruiz et al., 1994). However, over the last two decades, the application of PCR on *E. histolytica*/*E. dispar* samples initially identified by microscopy has led to the conclusion that the prevalence of *E. histolytica* in Africa was indeed overestimated (Kebede et al., 2003; Ben Ayed et al., 2008; Efunshile et al., 2015; Yimer et al., 2017).

The prevalence of *E. histolytica* in this study (7.5%) was higher than in most studies from African countries such as in Uganda (1.5%) (Morawski et al., 2017), Tanzania (2.9%) (Beck et al., 2008), Ethiopia (1.7%) (Yimer et al., 2017), Sudan (5.0%) (Saeed et al., 2015) and South Africa (4.1%) (Samie et al., 2020). However, the prevalence was lower than those reported from patients with gastrointestinal complaints in South Africa (15.6%) (Samie et al., 2006) and Egypt (10.3%) (Roshdy et al., 2017). In Kenya the prevalence of *E. histolytica* in this study was lower than (14.4–15%) reported previously (Easton et al., 2016; Mwendwa et al., 2017) but higher than (0.4–4.5%) in other studies (Matey et al., 2016; Kyany'a et al., 2019). The difference in prevalence could be due to the diagnostic tool applied for example Easton et al. (2016) used Real-time PCR which is more sensitive than conventional or nested PCR for detection and differentiation of *Entamoeba* spp. complex (Lau et al., 2013). Although the study by Mwendwa et al. (2017) shared the same study site with the current one, all the faecal samples were tested for *E. histolytica* by PCR while the present study applied *E. histolytica* specific PCR on *Entamoeba* spp. positive faecal samples following microscopic examination. The different study populations, geographical sites, environmental conditions, their socio-economic status and immune status could also explain the difference prevalence with other studies in Kenya.

The age group 21–30 years was the most infected with *Entamoeba* spp. including *E. histolytica* and is in agreement with previous studies in Kenya (Kamau et al., 2012; Kimosop et al., 2018). Populations sandwiching this age group recorded high *E. histolytica* infections in different countries for example 18–40 years in Yemen (Al-Areeqi et al., 2017) and 20–46 years in South Africa (Samie et al., 2006; Samie et al., 2020). This age-related prevalence of *E. histolytica* was also reported in Malaysia, where infections were more common in children below the age of 15 years (Shahrul Anuar et al., 2012). The age-dependency prevalence of *E. histolytica* is influenced by acquired immunity (Haque et al., 2006) and the interaction of this *Entamoeba* spp. and with host microbiome (Ngobeni et al., 2017; Leon-Coria et al., 2020). The age group 21–30 years was also shown to harbour the highest burden of other intestinal parasites in Kenya (Kipyegen et al., 2012). This age group in Kenya comprises of young adults who are likely buy food from the streets with low standards of hygiene and therefore exposing them to foodborne diseases. A recent study reported that buying food from the street as a common behaviour for residents of Mukuru slums and found significant association of this habit with salmonella disease (Mbae et al., 2020). This study found an inverse relationship between *E. histolytica* infections and diarrhea. The finding of positive association between *E. histolytica* infections and diarrhea (Samie et al., 2006; Yimer et al., 2017; Samie et al., 2020) or lack of association (Wumba et al., 2010) has been reported previously. A similar inverse relationship has been reported for *G. lamblia* in case-controls studies paired by age and sex in other sub-Saharan Africa counties including Côte d'Ivoire, Central African Republic, and Tanzania (Becker et al., 2015; Tellevik et al., 2015; Breurec et al., 2016). The cause of the inverse relationship between *Entamoeba* spp. infections and diarrhea could be due to infections with other parasitic agents. In this study 19 participants who had diarrhea but no *E. histolytica* infections were infected with *G. lamblia* (12), *A. lumbricoides* (5), a co-infection of *H. nana* and *H. diminuta* (1), *Cryptosporidium* spp. (1) and *Cyclospora* spp. (1). The cause of diarrhea in those negative for *E. histolytica* could also be other agents besides parasites such as viruses (Gikonyo et al., 2017) and bacterial infections (Kariuki et al., 2019) as reported before in residents of Mukuru informal settlement.

Previous studies in Kenya recorded lower prevalence of *E. dispar* than the present study, but were in agreement with this study that *E. dispar* was more common in patients than *E. histolytica* (Matey et al., 2016; Kyany'a et al., 2019). Although *E. dispar* is considered common among asymptomatic patients, it has been reported in symptomatic patients (Ximenez et al., 2010; Oliveira et al., 2015; Samie et al., 2020) including those with liver abscess (Dolabella et al., 2012). The proportion of participants infected with *E. dispar* and presented with or without diarrhea were comparable in this study (not statistically significant), however, it should be noted that majority of the *E. dispar* infections occurred as a co-infection with *E. histolytica* (86.3%).

This study failed to detect *E. moshkovskii* in patients with gastrointestinal symptoms and is consistent with previous findings in Mukuru slums (Mwendwa et al., 2017) and elsewhere in Kenya (Easton et al., 2016; Matey et al., 2016). However, *E. moshkovskii* was the most prevalent *Entamoeba* spp. detected in symptomatic and asymptomatic participants in Kenya (Kyany'a et al., 2019). The failure to detect *E. moshkovskii* in this study could be due to the inability by this nested PCR to amplify DNA from limited number of cysts of this species compared to those of *E. histolytica* and *E. dispar* (Khairnar and Parija, 2007; Lau et al., 2013). Indeed Hamzah et al. (2010) showed that a ten-fold amount of *E. dispar* and *E. moshkovskii* DNA (2 pg) was detectable by real-time PCR compared to that of *E. histolytica* (0.2 pg). Furthermore, a single round PCR assay needed double amount of DNA for the detection of *E. histolytica* and *E. moshkovskii* compared to that of *E. dispar* (Hamzah et al., 2006).

The failure by nested PCR to detect *Entamoeba* spp. DNA in microscopically-positive faecal samples could be due to the presence of PCR inhibitors that were not completely eliminated during DNA extraction. Another possible reason is limited amount of cysts or trophozoites in those samples that fell below the nested PCR detection limit. It is also postulated that faecal samples containing trophozoites alone compared to those with cysts are likely to fail on PCR due to their fast degradation. Lastly the cysts from those samples could belong to other *Entamoeba* spp. such as *E. polecki* which was not tested in this study, and confusion of macrophages with trophozoites or polymorphonuclears with cysts (Fotedar et al., 2007; Ngui et al., 2012).

The limitations of this study included 1) The examination of a single faecal sample instead of three-days consecutive samples is likely to underestimate the prevalence of *Entamoeba* complex because cysts or trophozoites are shed periodically (Nazer et al., 1993). 2) The use of microscopy for detection of *Entamoeba* spp. in faecal samples instead of nested PCR could also lower the actual prevalence of *Entamoeba* species given the limited sensitivity of microscopy. 3) The cause of diarrhea and abdominal discomfort could have been associated with other infections such viruses (Rotavirus, adenoviruses) and bacteria which were not diagnosed in this study. 4) The data on patients presenting with abdominal pain was not recorded and analyzed in the current study and this has been reported as a major symptom of amoebiasis (Samie et al., 2020).

In conclusion, this study identified *E. histolytica, E. dispar, E. coli* and *E. hartmanni* in patients seeking treatment for diarrhea and/or abdominal discomfort from two clinics in Mukuru informal settlements in Nairobi, Kenya. Further studies are needed on the occurrence of *Entamoeba* species in asymptomatic individuals who might act as reservoirs of *E. histolytica* and account for majority of amoebiasis infections. The study also highlights the need for differentiation of *E. histolytica* from other *Entamoeba* spp. by molecular tools for better management of amoebiasis to avoid unnecessary treatment for infections with non-pathogenic *Entamoeba* spp.

## Financial support

This study was funded through the Kenya Medical Research Institute, Internal Research Grants L0407.

## Declaration of Competing Interest

The authors declare that they have no known competing financial interests or personal relationships that could have appeared to influence the work reported in this paper.
